# Basal Cell Adenoma of the Parotid Gland: A Rare Disease Entity

**DOI:** 10.7759/cureus.36841

**Published:** 2023-03-29

**Authors:** Fayez A Alrohaimi, Farhan M Alanazi, Abdulaziz B Almutairi, Hisham M Almousa, Sultan M Alqahtani

**Affiliations:** 1 Otolaryngology - Head and Neck Surgery, Prince Sultan Military Medical City, Riyadh, SAU; 2 Otolaryngology - Head and Neck Surgery, Prince Mohammed Medical City, Jouf, SAU; 3 Medical Intern, College of Medicine, Majmaah University, Majmaah, SAU; 4 Medical Intern, College of Medicine, King Saud University, Riyadh, SAU

**Keywords:** parotid salivary gland, salivary gland neoplasm, parotid swelling, parotidectomy, basal cell adenoma

## Abstract

Neoplasms of the salivary glands are of rare incidence, have a vague presentation, and follow a complex long-term clinical course. Both minor and major salivary glands have been implicated in dysplastic transformation, with parotid gland tumors being the most notable. Most of these tumors are benign in nature and are typically diagnosed and classified based on their histopathological presentation. In this report, we exhibit a rare case of basal cell adenomas (BCA), localized to the right parotid gland, in a 69-year-old male patient. Volume acquisition computed tomography (CT) imaging of the region was obtained with and without contrast, with relative reconstruction in both the coronal and axial planes. A soft tissue mass of 5 cm in diameter was detected in the superficial lobe of the right parotid gland. Fine needle aspiration (FNA) with ultrasound guidance revealed a population of basaloid cells that is monomorphic with minimal nuclear atypia and scattered fibrillary matrix. Thereafter, the patient was treated with partial excision of the right parotid gland under general anesthesia, and the post-operative pathology report confirmed the diagnosis of basal cell adenoma. The patient was doing well post-operatively with no complaints and maintained routine clinic follow-ups.

## Introduction

Benign salivary gland neoplasms encompass an array of histopathological presentations and affect multiple sites in the head and neck region. The most commonly affected major salivary gland is the parotid gland, followed by the submandibular and sublingual glands, respectively. Among the minor salivary glands, upper lip neoplasms are the most frequently reported, followed by tumors in the buccal mucosa [1].

The World Health Organization (WHO) classifies benign salivary gland tumors into 11 different types, including basal cell adenoma (BCA). They describe BCA as a benign unspecified entity with an overall incidence rate of 1.1-3.7% [2].

Monomorphic basaloid epithelial cell proliferation constitutes the histopathological hallmark of BCA. Clinically, BCA is typically described as a slow-growing mass that is seldom symptomatic and is freely movable [3].

Adults between their fifth and seventh decades of life are most commonly affected. BCA carries a favorable prognosis in comparison with other salivary gland neoplasms, especially since recurrence is not common. Since BCA and basal cell adenocarcinoma share similar histopathological features, it can sometimes be difficult to distinguish them from one another. However, the latter generally presents with the invasion of the surrounding structures [4].

Here, we report a case of a parotid mass presenting as an asymptomatic swelling histopathologically identified as basal cell adenoma.

## Case presentation

We report a 69-year-old male, medically free and with no identifiable allergies, who was referred to our otorhinolaryngology (ENT) general clinic for the evaluation of a painless swelling in his right parotid region that had been present for a 10-year period. On further examination, the patient disclosed having no associated symptoms besides the noticeable swelling of his cheeks. There was no history of dyspnea or dysphagia. The patient denied any history of pain, skin color changes, numbness, facial weakness, xerostomia, odynophagia, or other obstructive symptoms.

On physical examination, the patient was vitally stable. A single, firm, non-tender swelling of his right parotid region was palpable. It was mobile and not fixed to the underlying structures. On examination of the neck lymph nodes, they were all non-palpable. The facial nerve proved intact, and the rest of the examination pertaining to the head and neck was unremarkable.

**Figure 1:**
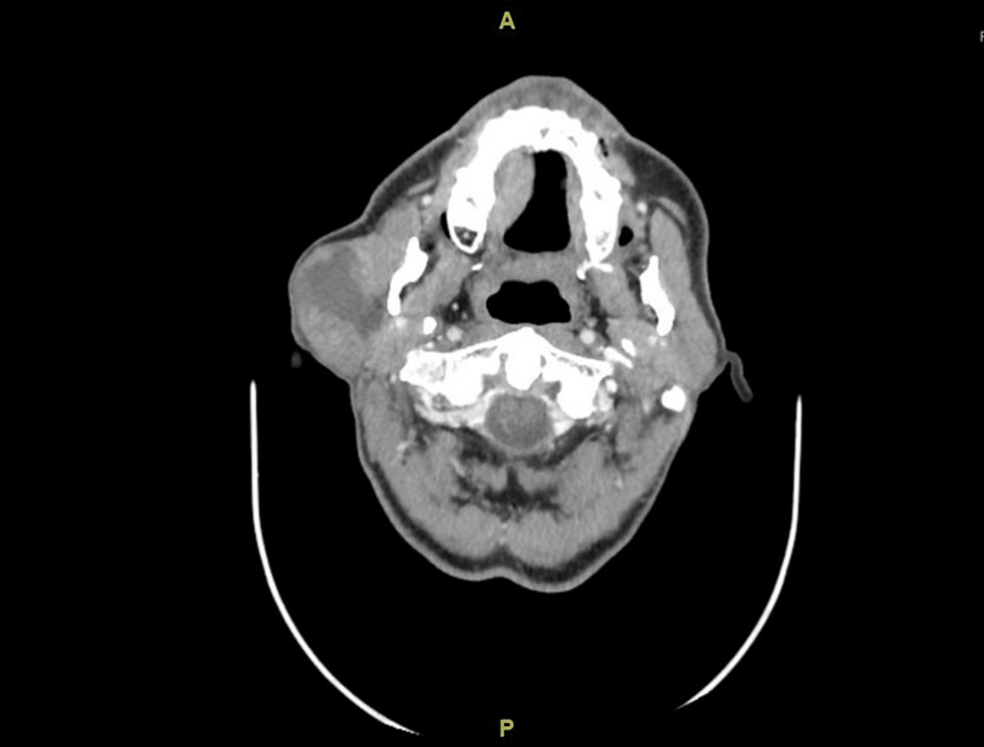
A contrast-enhanced axial CT scan shows a round, well-defined mass in the superficial lobe of right parotid gland with homogeneous enhancement.

**Figure 2:**
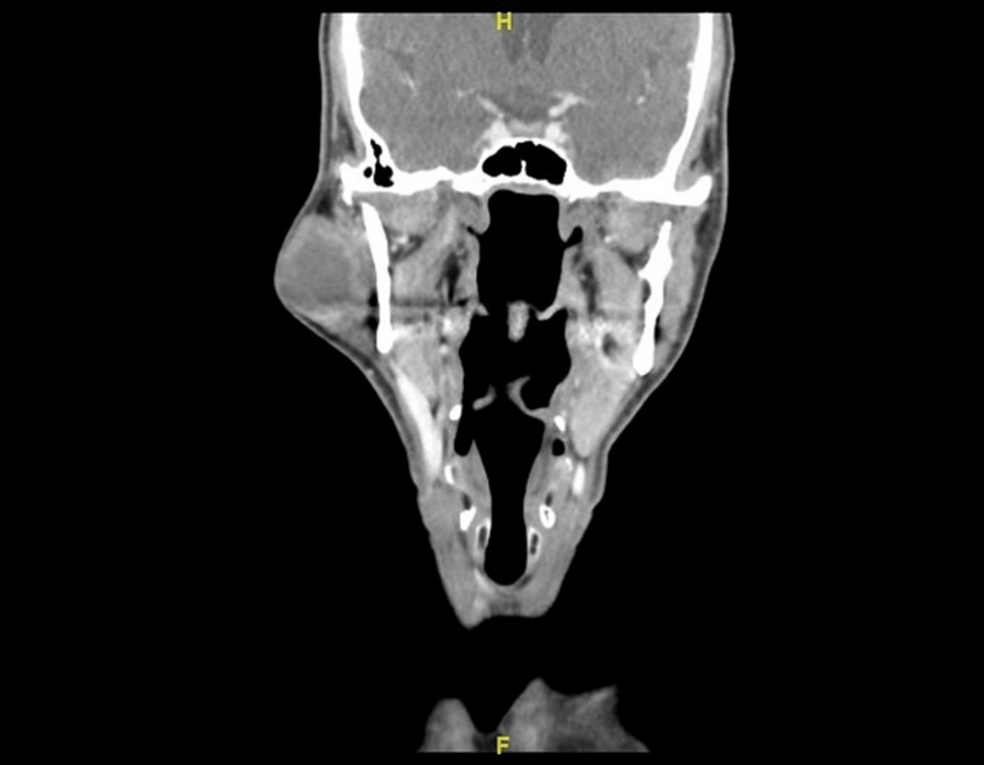
Coronal contrast-enhanced CT soft tissue window of the head showing a mass of soft tissue density in the superficial lobe of the right parotid gland.

On multiplanar CT scanning of the head and neck, a unilateral mass of soft tissue density in the right parotid gland arising from the superficial lobe was evident. The mass carried a mean diameter of 5 cm in its greatest dimension (Figures 1-2).

The patient was arranged for ultrasound-guided fine needle aspiration (FNA), with three passes of FNA being uneventful. FNA showed basaloid cells to have a monomorphic population with minimal nuclear atypia along with a scattered fibrillary matrix, without mitosis or any tumor necrosis. The features were suggestive of a cellular basaloid neoplasm.

**Figure 3:**
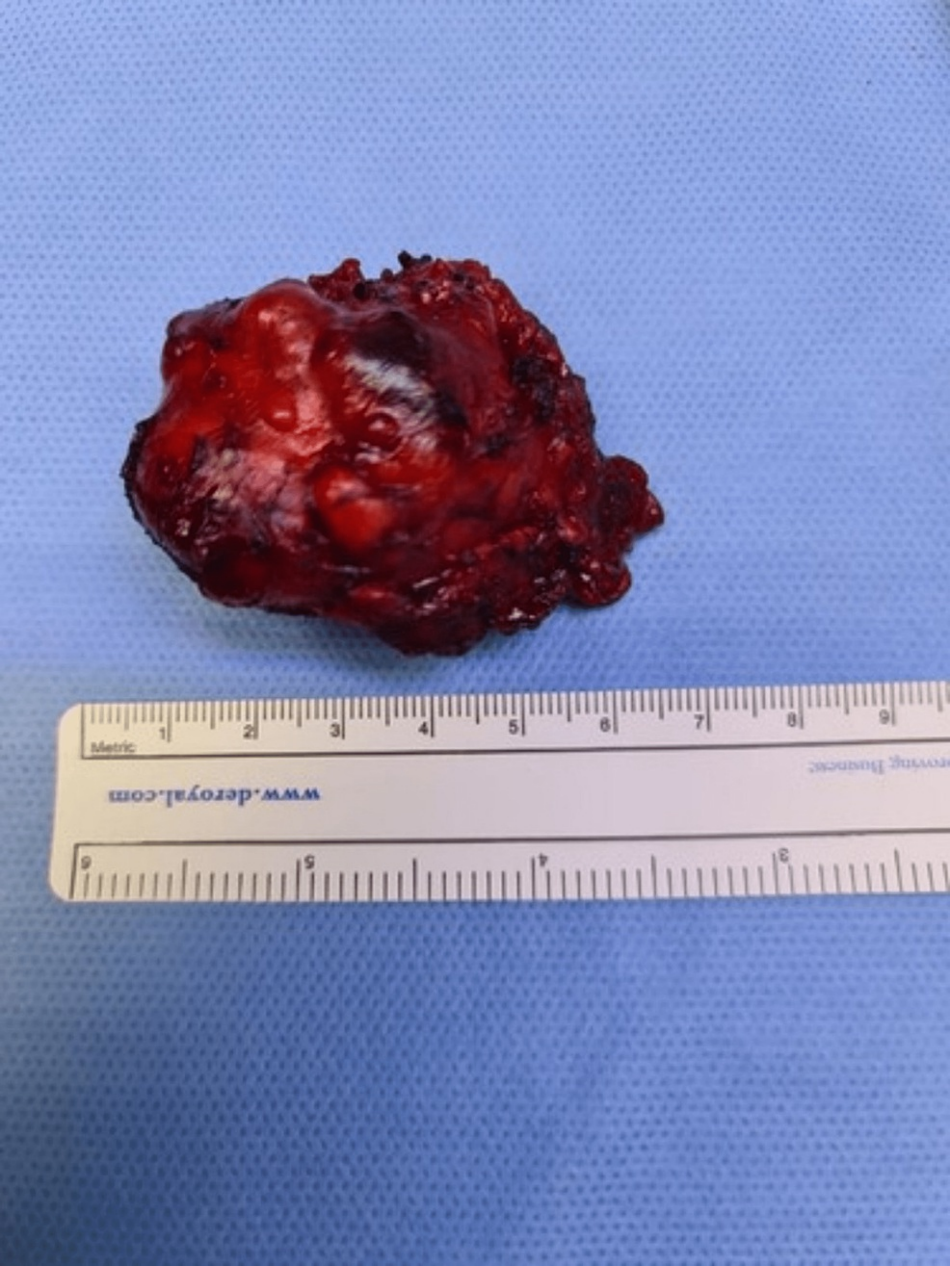
Postoperative excised specimen of the right parotid mass measuring 6.5 centimeters.

The treatment of choice was a right partial parotidectomy under general anesthesia. The surgery was uneventful, and the mass was sent for histopathological examination (Figure 3). The pathology report confirmed the mass to be consistent with a basal cell adenoma, with immunostaining showing both ductal and basal cell components that were positive for CKAE1/AE3 and P63, respectively. The patient was doing well post-operatively with no complaints and maintained routine clinic follow-ups.

## Discussion

The WHO salivary gland tumor classification recognizes the BCA of the salivary glands as an independent entity in its fifth edition [2]. The large majority of all parotid tumors are of benign nature, with pleomorphic adenoma being the most common type. On the other hand, monomorphic adenomas, including basal cell adenomas, are much less common and rarely reported. BCA can be of the solid, trabecular, membranous, or tubular type, and although recurrence is not uncommon, the membranous type was found to have a higher recurrence rate and a higher predilection toward malignant transformation than the other reported subtypes. This highlights the necessity of early recognition and therapeutic intervention [5]. BCA is typically slow-growing, firm, mobile, and painless, with a tendency to involve the superficial lobe. It also usually presents with a brownish-like color. The diagnosis relies on the histopathological examination of the obtained biopsy as the gold standard, although FNA is another acceptable alternative. However, for FNAs, physical access to the tumor must be secured first, and it is not more accurate than a biopsy. Uniform and regular basaloid cells upon histological evaluation of the tissue specimen are the defining characteristics of such a tumor.

The histological features in our case are consistent with those reported in the literature, showing a population of basaloid cells that are monomorphic with slight nuclear atypia associated with a dispersed fibrillary matrix [6].

Jang et al. reported three cases of BCA involving the parotid gland. Their aim was to describe the appearance of these tumors on computed tomography (CT) and magnetic resonance (MR) imaging. They came to the conclusion that all three studied cases shared the same imaging appearance of being well-circumscribed, having both solid and cystic or pure solid components, and enhancement upon contrast injection. However, not all of them were localized to the superficial lobe; two were located in the deeper portion of the parotid with protrusions toward the superficial lobe [7]. Our case imaging findings are consistent with what the literature has reported. The average reported age of patients presenting with basal cell adenomas is 57.7 years. In our case, the patient was slightly older than that. Delayed presentation and a vague clinical course are believed to be the reasons behind this delayed diagnosis.

## Conclusions

BCA of the parotid gland is a rare tumor with a wide list of differential diagnoses. Its ambiguous clinical picture as an asymptomatic swelling necessitates the need for early recognition, histopathological diagnosis, and therapeutic intervention. Challenges such as delayed presentation and loss of follow-up have made it necessary that physicians be aware of such implications for the health system and the patient's psychosocial status.
